# Acute Kidney Injury Associated With Urinary Stone Disease in Children and Young Adults Presenting to a Pediatric Emergency Department

**DOI:** 10.3389/fped.2020.591520

**Published:** 2020-11-30

**Authors:** Nicholas Farris, Rupesh Raina, Abhishek Tibrewal, Miraides Brown, Maria Colvis, Andrew Schwaderer, Kirsten Kusumi

**Affiliations:** ^1^Department of Pediatrics, Akron Children's Hospital, Akron, OH, United States; ^2^Division of Nephrology, Akron Children's Hospital, Akron, OH, United States; ^3^Division of Nephrology, Akron General Cleveland Clinic, Akron, OH, United States; ^4^Rebecca D. Considine Research Institute, Akron Children's Hospital, Akron, OH, United States; ^5^Kent State University, Kent, OH, United States; ^6^Division of Nephrology, Department of Pediatrics, Indiana University School of Medicine, Indianapolis, IN, United States; ^7^Northeast Ohio Medical University, Rootstown, OH, United States

**Keywords:** urolithiasis, kidney stones, AKI, pediatric, urinary stone disease (USD)

## Abstract

**Background:** Acute kidney injury (AKI) due to urinary stone disease (USD) is rare in adults; AKI rates in children with USD may be higher, and emerging data links stones to chronic kidney disease (CKD) development in adults.

**Methods:** This study is a retrospective analysis of USD patients at a single pediatric hospital system's emergency department (ED). Patients were initially identified by USD ICD codes; USD was then confirmed by imaging or physician documentation; patients had to have baseline creatinine (Cr) and Cr in the ED for comparison to be included. AKI was defined by Kidney Disease: Improving Global Outcomes (KDIGO), Acute Kidney Injury Network (AKIN), and Pediatric Risk, Injury, Failure, Loss, End Stage (pRIFLE).

**Results:** Of the 589 total visits, 264/589 (45%) had data to evaluate for AKI, 23% were AKI(+) and 77% were AKI(–). pRIFLE was most common (82%) and 18% were only positive by AKIN/KDIGO. AKI(+) were more likely to be younger (16.7 vs. 17.4 years, *p* = 0.046) and more likely to present with vomiting {odds ratio [OR] [95% confidence interval (CI)]: 2.4 [1.4–4.3], *p* = 0.002}; also, the proportion of AKI(+) was significantly higher in <18 vs. ≥18 years [26.9 vs. 15.5%, *p* = 0.032, OR (95% CI): 2.0 (1.1–3.9)]. Urinary tract infection (UTI) and obstruction rates were similar between groups. AKI(+) patients had a significant OR <1 suggesting less risk of receiving non-steroidal anti-inflammatory drugs (NSAIDs); however, 51% of them did receive NSAIDs during their ED encounter. AKI(+) patients were more likely to require admission to the hospital (53 vs. 32%, *p* = 0.001).

**Conclusion:** We have demonstrated a novel association between USD-induced renal colic and AKI in a group of young adults and children. AKI(+) patients were younger and were more likely to present with vomiting. AKI(+) patients did not have higher rates of obstruction or UTI, and 51% of AKI(+) received NSAIDs.

## Introduction

Urinary stone disease (USD) is common in the United States, affecting 1 in 10 adults, and is becoming more common in the pediatric population (1). The incidence of pediatric USD has increased by 4–6% annually, and the number of stone-forming adolescents has doubled in the last 20 years (1, 2). While urinary stones are notorious for causing severe pain and renal colic, morbidity has traditionally been linked to urinary tract obstruction and/or infection (3). Acute kidney injury (AKI) is defined by an abrupt decrease in renal function and has been strongly linked to increases in morbidity and mortality (4–7). USD-associated AKI is most often attributed to the obstruction of a solitary kidney or both kidneys simultaneously and occurs in 1–2% of adult stone formers (8–11). AKI in pediatric USD formers has not been well-described, but there is some evidence that children may be more susceptible with rates as high as 30% (12).

In adults, increases in creatinine (Cr) as small as 0.3 mg/dl have been linked to increased mortality; furthermore, AKI can increase hospital care costs and length of stay (5, 6, 13, 14). While AKI is uncommon in adult USD formers, there is evidence that they are at increased risk for development of chronic kidney disease (CKD) independent of other comorbidities such as diabetes and hypertension (1, 15–18). If pediatric USD formers develop AKI more frequently, they may be at greater risk for long-term renal sequelae as children have higher stone recurrence rates compared to adults due to their greater prevalence of stone metabolic risk factors (19). Thus, younger patients cared for in a children's hospital may constitute a previously unrecognized high-risk group for AKI with potential for long-term CKD development. Therefore, the objective of this study was to evaluate acute renal colic patients in a pediatric emergency department (ED) for AKI.

## Materials and Methods

### Study Design

This study was a retrospective review with approval by the Akron Children's Institutional Review Board (ID 1205631-7). Electronic medical records were reviewed for all patients under 25 years at a single freestanding pediatric hospital system identified by ICD coding from January 2008 to December 2017. The ICD 10 codes included the following: N20, N20.1, and N20.9 and ICD9: 592, 592.1, and 592.9 (calculus of kidney, ureter, and lower urinary tract, as well as urinary calculus, unspecified). All charts were reviewed to confirm diagnosis of urinary stones, and patients were only included if they either had (1) radiographic imaging positive for stones [renal ultrasound or computed tomography (CT)] or (2) documentation by an attending nephrologist or urologist of kidney stone disease. Patient charts were then reviewed for ED visits due to stone-associated renal colic. Visits were included if they either had (1) positive imaging (renal ultrasound or CT) of stones at that visit or (2) documentation by an attending physician that presenting symptoms were due to urinary stones.

### Data Collection

The data collected included anthropometrics at the time of ED visit (age, sex, weight, height, BMI, and corresponding *Z*-scores), presenting clinical symptoms (flank pain, abdominal pain, nausea, vomiting, hematuria, and/or dysuria), serum chemistry, urinalysis, cultured urine organisms, and imaging (renal ultrasound and CT). Abdominal X-rays of the kidney, ureter, and bladder (KUB) were not included as local practice patterns do not routinely utilize KUB as a screening tool in the ED, without also obtaining other imaging, due to the inability of KUB to evaluate patients for obstruction. Any urine culture with an organism growing >100,000 colony forming units was considered positive, even if the organism is not considered a traditional urinary infectious organism. Treatment in the ED including medications [intravenous fluids (IVF), pain medications, anti-emetics, alpha-agonists, and antibiotics] as well as discharge medications were identified. Disposition (hospital admission or discharge) were also reported.

### AKI Definition

AKI was defined utilizing three well-established pediatric criteria: the Kidney Disease Improving Global Outcomes (KDIGO), the Acute Kidney Injury Network (AKIN), and the Pediatric Risk, Injury, Failure, Loss, End Stage Renal Disease (pRIFLE) criteria (Table 1). The temporal and urine output criteria for AKI were not used due to the retrospective analysis and short-term nature of ED care. ED visits were designated as either able or unable to evaluate for AKI based on availability of data including height measurement and serum Cr levels. Baseline Cr and height measurements were included from outpatient visits, if available, from within the following time ranges dependent on the patients' age at the time of their ED visit: infants within 3 months, 2–6 years old within 6 months, 6–11 years within 8 months, 11–14 years within a year, and 14+ within 2 years. If multiple Cr measurements were available, baseline Cr was defined as the lowest value within the above age-defined time range. The glomerular filtration rate (GFR) was calculated using the bedside Schwartz formula (20). Any patient without available baseline serum Cr level was categorized as unable to evaluate for AKI and excluded from subsequent analysis.

**Table 1:** AKI definitions.

**AKI criteria**
pRIFLE	Stage 1 (Risk)	eGRF decreased by 25%
	Stage 2 (Injury)	eGRF decreased by 50%
	Stage 3 (Failure)	eGRF decreased by 75% or eGFR <35 ml/min per 1.73 m^2^
KDIGO	Stage 1	Increase in Cr of ≥50% or absolute increase in Cr of 0.3 mg/dl
	Stage 2	Increase in CR of ≥100%
	Stage 3	Increase in Cr of ≥200%
AKIN	Stage 1	Increase in Cr of ≥50% or absolute increase in Cr of 0.3 mg/dl
	Stage 2	Increase in Cr of ≥100%
	Stage 3	Increase in Cr of ≥200%

*Cr, creatinine; eGFR, estimated glomerular filtration rate*.

### Statistical Analysis

Patient data were analyzed by ED visit and the visits were then compared: AKI by the criteria of pRIFLE, KDIGO, or AKIN [AKI(+)] vs. those without AKI [AKI(–)]. Comparisons of the AKI(+) vs. AKI(–) groups were made with Chi-square or Fisher's exact test for categorical variables and Student *t*-tests or Wilcoxon Rank-Sum tests were performed for continuous variables. The Hodges–Lehmann Estimator and odds ratio confidence intervals were calculated where appropriate. Statistical analyses were completed using SAS (version 9.4; SAS Institute Inc., Cary, NC, USA). All statistical tests were two-tailed with significance defined as *p* <0.05. The Venn diagram was generated by using Venny 2.1 online software (21).

## Results

### Demographics

**Figure 1:**
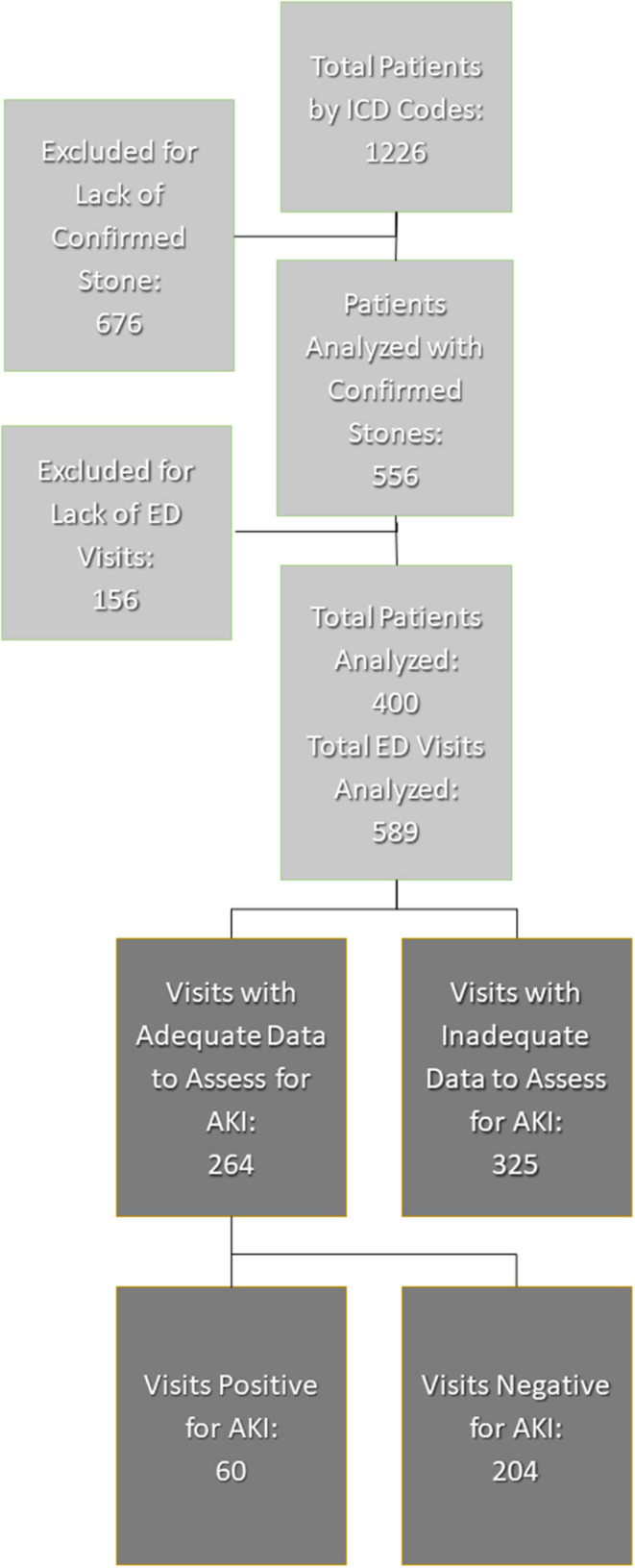
Inclusion and exclusion of patient visits. AKI, acute kidney injury; ED, emergency department.

A total of 1,226 patients, aged 1–25 years old, had ICD codes for kidney stones while 670 patients were excluded due to insufficient data to confirm urinary stone disease. Another 156 patients were excluded due to lack of ED visit for acute renal colic. Overall, there were 400 unique patients with 589 total visits that met the inclusion criteria for analysis (Figure 1).

### AKI Rates

Of the 589 total visits, 264/589 (45%) had adequate data to evaluate for AKI corresponding to 146 unique patients. All these patients had both baseline creatinine as well as creatinine at ED. Among the included patients, 86/146 (59%) had one visit, 44/146 (30%) had 2–3 visits, and 16/146 had more than three visits. A total of 60/264 (23%) visits met criteria for AKI while 204/264 (77%) did not.

**Figure 2:**
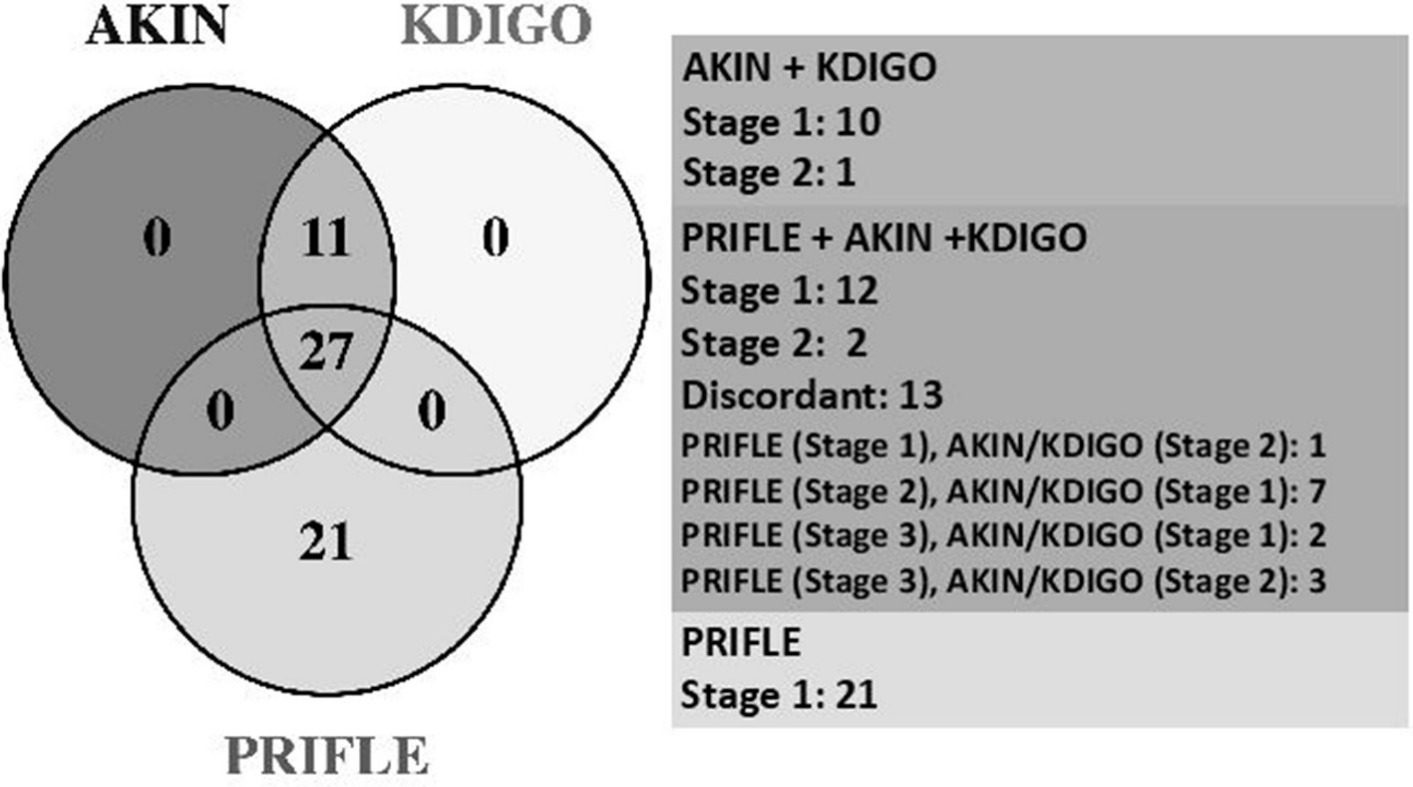
Comparative stages of AKI. AKIN, Acute Kidney Injury Network; KDIGO, Kidney Disease Improving Global Outcomes; PRIFLE, Pediatric Risk, Injury, Failure, Loss, End Stage Renal Disease.

Of all visits with AKI, all three criteria (pRIFLE, AKIN, and KDIGO) were met with 28/60 (47%), and pRIFLE was the most frequently met AKI criterion with 49/60 (82%) of visits compared to 38/60 (63%) of visits for AKIN and KDIGO. However, only 21/60 (35%) met pRIFLE criterion and only 11/60 (18%) visits met the AKIN/KDIGO criteria due to the former's inclusion of an increase in serum Cr > 0.3 mg/dl from baseline. The distribution of AKI diagnoses by each criterion is shown in Figure 2.

### Comparison of AKI(+) and AKI(–) Groups

Table 2 displays demographic and clinical characteristics for AKI(+) vs. AKI(–) groups. We included all patient visits for USD in the ED of our pediatric hospital system including young adults; a total 167 patients were <18 years with 45 (26.9%) AKI(+) vs. 122 (73.1%) AKI(–) vs. a total of 97 patients ≥18 years with 15 (15.5%) AKI(+) vs. 82 (84.5%) AKI(–). The oldest patient in our cohort was 24 years. The proportion of AKI(+) was observed to be significantly higher among those aged <18 years as compared to those aged ≥18 years {26.9 vs. 15.5%, *p* = 0.032, odds ratio [OR] [95% confidence interval (CI)]: 2.0 [1.1–3.9]} resulting in slightly younger age profile among those with AKI(+) vs. AKI(–) patients (median: 16.7 vs. 17.4 years, *p* = 0.045). All other anthropometric variables were similar in both groups. Also, AKI(+) patients were 2.5 times more likely to present with complaints of vomiting [OR (95% CI): 2.5 (1.4–4.4), *p* = 0.002] and 3.2 times more likely to complain of fever [OR (95% CI): 3.2 (1.1–9.3), *p* = 0.049]. Patients with AKI(+) had higher blood urea nitrogen (BUN) (13 vs. 12, *p* = 0.007) and Cr levels at ED (0.9 vs. 0.7, *p* < 0.001) vs. AKI(–) patients on serum testing. Also, patients with AKI(+) had lower GFR at ED (72.3 vs. 96.2, *p* < 0.001) and higher percentage decrease in GFR from baseline to ED (34 vs. 6%, *p* < 0.001) vs. AKI(–) patients. Although AKI(+) patients demonstrated slightly lower sodium levels {median [interquartile ranges (IQR)]: 138 [136.3, 139] vs. 138 [137–140], *p* = 0.043}, due to the retrospective nature of the study, it is not clear when these labs were obtained in relation to IVF therapy. Despite a higher incidence of complaints of fever on presentation among AKI(+) patients [AKI(+) 11.7% vs. AKI(–) 3.9%, *p* = 0.049), there was no significant difference between the groups' leukocyte esterase and nitrite testing on urinalysis. Furthermore, 65–70% of AKI(+) and AKI(–) groups had formal urine cultures with similar rates of positive urine culture growth. None of our AKI(+) patients had a solitary kidney, and we did not find a significant difference in rates of unilateral or bilateral obstruction between AKI(+) and AKI(–) groups (unilateral 66.7 vs. 57.5%, *p* = 0.19; bilateral 1.8 vs. 3.8%, *p* = 0.46).

**Table 2:** Comparison of AKI(+) and AKI(–) groups.

**Variables**		**AKI(+)**		**AKI(–)**		***p*-value**
		***n***	**Median (IQR) or Percentage**	***n***	**Median (IQR) or Percentage**	
Anthropometrics	Age (years)	60	16.65 (12.8–18)	204	17.4 (15.4–18.8)	**0.04**
	Female	40	66.7%	120	58.8%	0.27
	Weight (kg)	60	61.7 (49.7–73.6)	203	61.2 (51.3–76.2)	0.43
	Weight Z-score	54	0.6 (−0.3–1.5)	177	0.5 (−0.3–1.3)	0.93
	Height (cm)	56	160 (152.3–167)	149	163 (154.5–168.7)	0.24
	Height Z-score	42	−0.39 (−0.9–0.4)	95	−0.12 (−0.8–0.5)	0.55
	BMI	55	22.2 (19.5–28.1)	148	23.54 (19.9–29.2)	0.76
	BMI Z-score	51	0.8 (−0.2–1.8)	128	0.91 (−0.1–1.6)	0.95
Presenting symptoms	Abdominal pain	39	65%	117	57.4%	0.29
	Flank pain	39	65%	148	72.6%	0.26
	Nausea	28	46.7%	80	39.2%	0.3
	Vomiting	35	58.3%	74	36.3%	**0.002**
	Fever	7	11.7%	8	3.9%	**0.04**
	Gross hematuria	12	20%	33	16.2%	0.49
	Dysuria	13	21.7%	37	18.1%	0.54
Chemistries	Sodium	60	138 (136.3–139)	203	138 (137–140)	**0.04**
	Potassium	60	3.8 (3.6–4)	203	3.9 (3.7–4.2)	0.08
	Chloride	60	104 (102–106)	203	105 (103–106)	0.18
	Bicarbonate	59	23 (22–25)	202	24.15 (22.1–25.5)	0.07
	BUN	60	13 (11–15)	203	12 (10–14)	**0.007**
	Creatinine baseline	60	0.59 (0.42–0.7)	204	0.7 (0.59–0.73)	** <0.001**
	Creatinine ED	60	0.9 (0.7–1.1)	204	0.7 (0.6–0.8)	** <0.001**
	Calcium	60	9.4 (9.1–9.68)	198	9.4 (9.1–9.6)	0.85
	GFR baseline	56	116.7 (99.3–145.8)	148	100.43 (92.1–118.2)	** <0.001**
	GFR ED	56	72.3 (62.9–89.2)	148	96.2 (83.8–110.5)	** <0.001**
	Absolute change in GFR	56	−36.6 (−54.3–−28.7)	148	−5.6 (−13.7– 0)	** <0.001**
	% change in GFR	56	−34 (−42 to −27)	148	−6 (−13–0)	** <0.001**
Urinalysis	Specific gravity	56	1.02 (1.02–1.03)	193	1.02 (1.02–1.03)	0.66
	Leukocyte esterase (+ or trace)	12	21.8%	29	15.4%	0.27
	Nitrite (+)	1	1.8%	18	9.6%	0.06
	Hemoglobin (+)	45	81.8%	151	78.6%	0.61
	Protein (+)	23	41.8%	74	39.2%	0.72
	Positive urine cultures	4	10%	15	11%	1
Imaging	Imaging obtained (RUS or CT)	57	95%	186	91.2%	0.34
	CT scan	36	64.3%	128	69.9%	0.42
	Renal ultrasound	20	35.7%	52	28.7%	0.32
	Unilateral obstruction	38	66.7%	107	57.5%	0.19
	Bilateral obstruction	1	1.8%	7	3.8%	0.46
Treatment	IV fluids	53	88.3%	177	86.8%	0.75
	Anti-emetics	28	46.7%	108	52.9%	0.39
	Alpha-blockers	5	8.3%	8	3.9%	0.18
	NSAIDs	33	55%	141	69.1%	**0.04**
	Tylenol	4	6.7%	8	3.9%	0.48
	Opiates	29	48.3%	105	51.5%	0.67
Discharge	Phenazopyridine (Pyridium)	0	0%	1	0.5%	1
	Tylenol	9	15%	34	16.7%	0.76
	Opiates	14	23.3%	70	34.3%	0.11
	Ibuprofen	7	11.7%	39	19.1%	0.18
	Ondansetron (Zofran)	10	16.7%	32	15.7%	0.86
	Alpha-blockers	12	20%	49	24%	0.52
	Ketorolac (Toradol)	2	3.3%	19	9.3%	0.18
Admission	Admitted from ED	30	53.6%	59	30.4%	**0.001**

*Continuous variables are presented as median with inter-quartile ranges (IQR), categorical are presented as percentages. Alpha-blockers included Tamsulosin, Alfuzosin, Terazosin, Doxazosin; NSAIDs included Ibuprofen or ketorolac. Bold indicates a significant p-value*.

### AKI Documentation by ED

**Figure 3:**
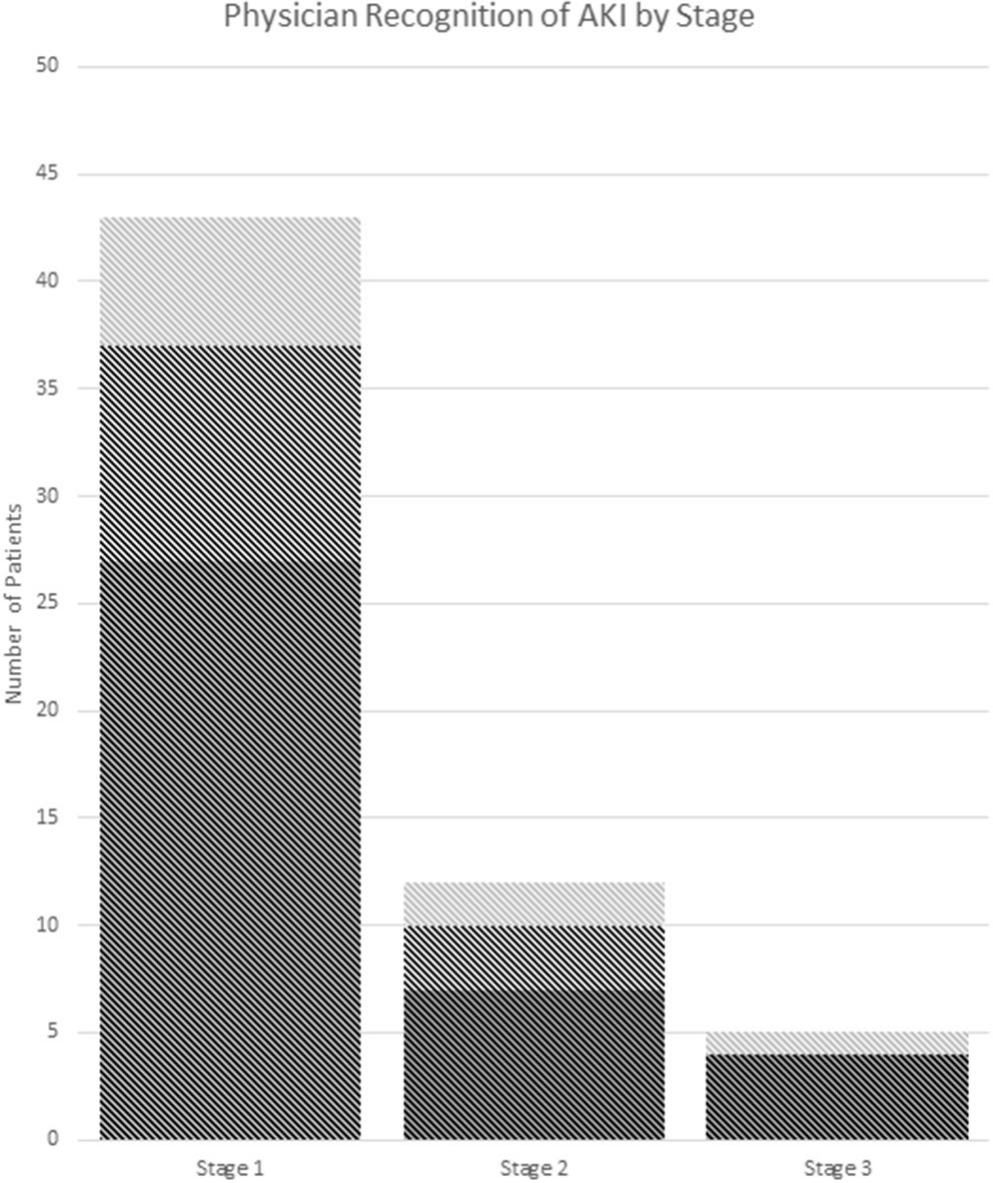
Recognition of AKI by stage. **(A)** Dark gray: no documentation. **(B)** Medium gray: documentation of abnormal labs. **(C)** Light gray: documentation of the presence of AKI or elevated Cr in assessment and/or plan. AKI stage is chosen by the highest stage met of any the pRIFLE, AKIN, or KDIGO criteria.

Thirty seven percent (22/60) of AKI(+) visits had documented recognition of their abnormal renal function in the chart with no significant difference between criteria used for defining AKI (Table 3). Of these visits, 59% (13/22) included lab values in their physician's note without mention of Cr levels being abnormal, 23% (5/22) included notation of elevated Cr in the physician assessment, and 18% (4/22) included notation of AKI (Figure 3).

**Table 3:** Comparison between AKI criteria and documented recognition of AKI.

**Criteria-based AKI**	**Documented recognition of AKI**		***p-*value**
	**Yes**	**No**	
pRIFLE/KDIGO/AKIN	10 (45.5%)	18 (47.4%)	0.347
KDIGO/AKIN	6 (27.3%)	5 (13.2%)	
pRIFLE	6 (27.3%)	15 (39.5%)	
Total	22 (100%)	38 (100%)	

### Treatment

**Figure 4:**
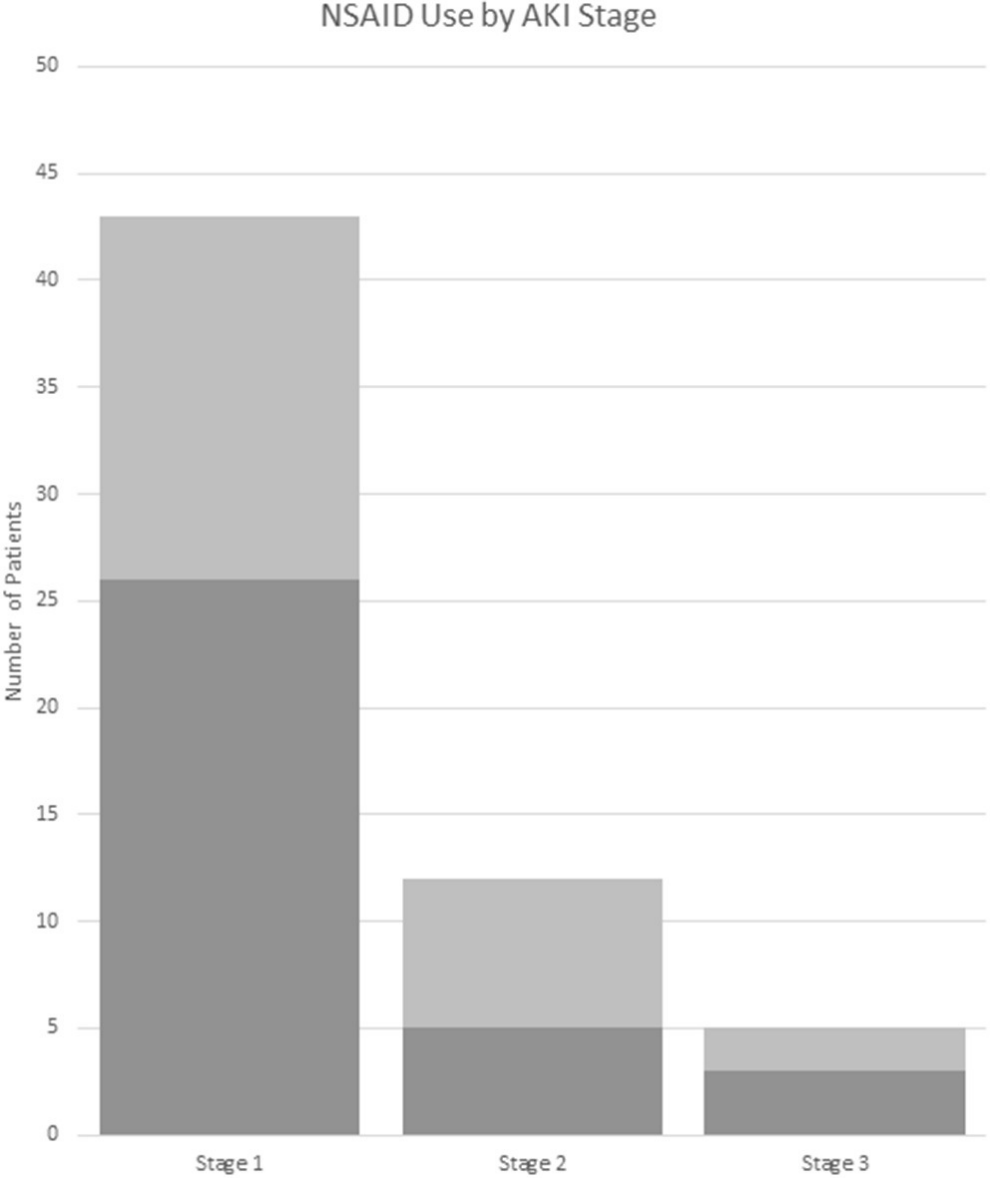
NSAID use by AKI stage. Dark gray: received NSAIDs. Light gray: did not receive NSAIDs. AKI stage is chosen by the highest stage met of any the pRIFLE, AKIN, or KDIGO criteria. NSAID, non-steroidal anti-inflammatory drug.

Of the treatments received in the ED, AKI(+) patients were slightly less likely to receive non-steroidal anti-inflammatory drugs (NSAIDS) [55 vs. 69%, *p* = 0.043; OR (95% CI): 0.55 (0.30–0.98)] as compared to AKI(–) patients (Table 2, Figure 4). However, AKI(+) patients were 2.6 times more likely to require admission to the hospital from ED [54 vs. 30%, *p* = 0.001, OR: 2.6 (1.4–4.9)]. There were no statistically significant differences in the treatments prescribed at discharge between AKI(+) and AKI(–) patients.

## Discussion

To our knowledge, this is the first study to assess a pediatric emergency department cohort for AKI associated with urinary stone-associated acute renal colic. Specifically, we have identified that AKI could only be assessed in less than half of ED visits due to insufficient data; of those visits with sufficient data, 23% of visits met the criteria for AKI. Most frequent missing data included serum Cr levels from the ED visit, height measurements, or baseline Cr for comparison. Pediatric AKI studies have typically focused on critically ill populations or those children requiring dialysis, thus making our analysis unique (8, 22–26). Additionally, large cross-sectional pediatric cohort studies have investigated inpatient populations and reported sepsis/shock, respiratory failure, liver or heart disease, bone marrow transplant, hemolytic-uremic syndrome, and nephrotoxic medications as the most common causes of pediatric AKI (25, 27–29). While urinary stones are noted in multiple studies, they are not considered a major cause of pediatric AKI; however, Jamal et al. found that urinary stones accounted for 30% of AKI in a single-center Pakistani cohort (12).

Notably, there is a paucity of data evaluating less severely ill patients for AKI in a children's hospital ED or outpatient setting. Furthermore, studies which did include ED patients are limited to those where patients being studied were subsequently admitted to the hospital (30). While the direct connection between AKI and development of CKD remains somewhat controversial, many recognize that milder episodes of AKI may contribute to long-term loss of GFR (4, 31). Given that adult urinary stone formers are at increased risk for CKD independent of established risk factors, this evidence of under-recognized AKI in urinary stone-forming children is particularly concerning (17, 18). Thus, while young adults and children with urinary stones have not previously been considered a high-risk population for AKI, we assert that urinary stone-associated AKI in this group is likely under-recognized and should be better addressed to prevent development of future CKD.

The high prevalence of AKI in our cohort was identified despite the fact that >50% of visits lacked the necessary data to assess for AKI. pRIFLE was the most frequently met definition for kidney injury; however, 18% of stone patients with AKI exclusively met the AKIN/KDIGO criteria. Thirty-five percent of patients would be missed by utilizing AKIN/KDIGO alone (Figure 2). The lack of concordance again highlights the need for greater consensus in the medical community regarding AKI criteria. The lack of an agreed-upon AKI definition limits researchers' ability to truly assess AKI prevalence and design of studies aimed at improving treatment and prevention (32). This is especially important as we begin to understand how common milder degrees of AKI are in pediatric populations and how AKI can impact long-term kidney function, including the development of CKD (31, 33, 34).

We demonstrated that younger urinary stone formers may be at greater risk for AKI based on a lower median age of AKI(+) vs. AKI(–) patients and that children have an odds ratio of 2 for suffering from AKI compared to those ≥18 years. This could potentially be due to the decreased probability of spontaneous stone passage in younger children leading to obstruction, which has traditionally been the main mechanism thought to drive stone-associated AKI (9, 11). However, we did not find a significant difference in rates of unilateral or bilateral obstruction between AKI(+) and AKI(–) groups (unilateral 66.7 vs. 57.5%, *p* = 0.22; bilateral 1.8 vs. 3.8%, *p* = 0.68), and none of our AKI(+) patients had a solitary kidney. Although, our assessment of obstruction was based on radiologist's official read including hydronephrosis or hydroureter, rather than the formal Mag-3 Renal Scan. Nonetheless, this preliminary data does raise the question of whether additional factors are diving AKI. In addition, we demonstrated that a significantly higher number of patients with AKI presented with complaints of vomiting (58.3 vs. 36.3%, *p* < 0.002). This prevalence of vomiting in the AKI(+) group is likely an indirect marker for pre-renal azotemia, a well-known cause of kidney injury in children. However, we did not find a significant difference between the specific gravity of urine samples in our AKI(+) vs. AKI(–) patients. This could be due to variability when urine samples were obtained in relation to IVF therapy, as patients who were significantly dehydrated may not have been able to provide a urine sample until they have already received IV hydration. AKI(+) patients were also more likely to complain of fever at presentation (11.7 vs. 3.9%, *p* = 0.049); however, there was not a significantly higher rate of positive urine culture in the AKI group, and thus, pyelonephritis is unlikely to be driving AKI in our population. Recently published evidence of a pro-inflammatory urinary tract environment in non-UTI USD adolescent patients could potentially account for complaints of fever and serve as a risk factor for AKI in this population (35).

ED management between groups was similar and most often included IVF, pain, and nausea control. Although AKI(+) patients had a significant OR <1, suggesting less risk of receiving NSAID compared to AKI(–) patients, 51% of them did receive NSAIDs during their ED encounter. This may be due to the low recognition of AKI in these patients by medical providers. Only 37% of AKI(+) patients had documentation in their chart of abnormal Cr levels, and 22% of these were counted due to the inclusion of labs in the physician's note. However, it is unclear if the abnormal nature of the Cr was recognized in these patients. More than 60% of AKI(+) patients did not have any documentation in their charts, and it is likely that their kidney injury was not recognized by the treating physician. Finally, we noted that children with AKI were more likely to require admission to the hospital. This higher admission rate is likely due to the presenting symptoms associated with AKI, such as vomiting, and the fact that these patients represent a more severely ill section of the pediatric stone cohort.

Limitations of this study include that data collection was from a single tertiary care pediatric hospital system in the Midwest; therefore, the practice patterns of this single care network may limit generalizability. Due to the retrospective nature of the study, data collection required reliance on electronic medical records for AKI assessment. Furthermore, estimating glomerular filtration rate (eGFR) from serum Cr levels utilizing the bedside Schwartz equation can result in differences in GFR estimates that are due to variations other than changes in kidney function in non-CKD children and adolescents (36). In non-CKD cohorts, changes in Cr may be representative of differences in growth, protein intake, or muscle mass rather than changes GFR. Thus, the changes in eGFR found in our cohort may be attributed, at least in part, to variation in creatinine-based GFR estimation. Implicit in this study design is our inability to accurately describe the timing of blood draws in relation to IVF administration, which has made assigning clinical meaning to laboratory findings such as small (but statistically significant) differences in serum sodium difficult and may have also influenced other laboratory findings such as urine specific gravity. While there are administration times documented by nurses in the medication administration record, we were unable to confirm how accurate this documentation was in relation to actual patient care. We were not able to utilize AKI criteria based on urine output due to the short nature of emergency room visits and could potentially miss patients who may have met criteria in a hospital setting. In addition, 55% of visits were lacking data for accurate AKI analysis, and approximately half of these did not have a serum Cr measurement at that ED visit. This could represent a systematic selection bias toward finding AKI in sicker patients as those patients which appeared more ill would be more likely to undergo laboratory evaluation. However, multiple patients could not have their GFR assessed due to the lack of height measurements, a limitation less likely to be affected by presenting illness severity. Even for patients with data to assess for AKI, the height was often obtained from outpatient visits within the defined age-based time range, again highlighting the difficulty in accurate recognition of AKI in the ED. Further complicating patient assessment was that the Cr obtained at that ED visit often represented the only available measurement for a given patient and thus limited physician's ability to compare multiple Cr measurements. AKI was recognized in a portion of the study population due to a lower post-visit Cr found at outpatient follow-up.

In this study, we demonstrated a novel association between urinary stone-induced renal coli and AKI in a group of young adults and children. This is of particular concern given the increasing number of children and adolescents affected by urinary stone disease and the known association of urinary stones with the development of CKD in adults (17, 18). Previous reports of urinary stone-associated AKI have been linked to urinary tract obstruction; however, our data suggests that dehydration may play a significant role. In addition, the poor recognition of milder stages of AKI by medical professionals and the reliance on NSAIDs for pain control need to be addressed. The lack of agreement on the definition of pediatric AKI and debate over whether AKI is truly causal for CKD limit our ability to draw a strong conclusion from retrospective data. However, this study does provide evidence that AKI in urinary stone-forming children and young adults is an area in need of greater research and attention. Thus, the potential for future CKD in a pediatric population who may develop recurrent mild episodes of AKI is concerning and should be addressed to improve their long-term care.

## Data Availability Statement

The raw data supporting the conclusions of this article will be made available by the authors, without undue reservation.

## Ethics Statement

The studies involving human participants were reviewed and approved by Akron Children's Hospital IRB. Written informed consent from the participants' legal guardian/next of kin was not required to participate in this study in accordance with the national legislation and the institutional requirements.

## Author Contributions

NF and MC: study design and data analysis and acquisition. RR: data analysis/interpretation and mentorship. AT and MB: data analysis/interpretation and statistical analysis. AS: research idea, study design, and statistical analysis. KK: research idea, study design, data analysis and acquisition, supervision/mentorship, took responsibility that this study has been reported honestly, accurately, transparently, and accepts accountability for the overall work by ensuring that questions pertaining to the accuracy or integrity of any portion of the work were appropriately investigated and resolved. All authors contributed to the article and approved the submitted version.

## Conflict of Interest

The authors declare that the research was conducted in the absence of any commercial or financial relationships that could be construed as a potential conflict of interest.
